# Cardiac troponins may be irreversibly modified by glycation: novel potential mechanisms of cardiac performance modulation

**DOI:** 10.1038/s41598-018-33886-x

**Published:** 2018-10-31

**Authors:** Johannes V. Janssens, Brendan Ma, Margaret A. Brimble, Jennifer E. Van Eyk, Lea M. D. Delbridge, Kimberley M. Mellor

**Affiliations:** 1 Department of Physiology, University of Melbourne, Melbourne, Australia; 2 Advanced Clinical Biosystems Research Institute, Smidt Heart Institute, Cedars-Sinai Medical Center, Los Angeles, USA; 3 Department of Chemical Sciences, University of Auckland, Auckland, New Zealand; 4 Department of Physiology, University of Auckland, Auckland, New Zealand; 5 Auckland Bioengineering Institute, University of Auckland, Auckland, New Zealand

## Abstract

Dynamic movements of the cardiac troponin complex are an important component of the cardiac cycle. Whether cardiac troponins are subjected to irreversible advanced glycation end-product (AGE) modification is unknown. This study interrogated human and rat cardiac troponin-C, troponin-I and troponin-T to identify endogenous AGE modifications using mass spectrometry (LC-MS/MS). AGE modifications were detected on two amino acid residues of human troponin-C (Lys_6_, Lys_39_), thirteen troponin-I residues (Lys_36_, Lys_50_, Lys_58_, Arg_79_, Lys_117_, Lys_120_, Lys_131_, Arg_148_, Arg_162_, Lys_164_, Lys_183_, Lys_193_, Arg_204_), and three troponin-T residues (Lys_107_, Lys_125_, Lys_227_). AGE modifications of three corresponding troponin-I residues (Lys_58_, Lys_120_, Lys_194_) and two corresponding troponin-T residues (Lys_107_, Lys_227_) were confirmed in cardiac tissue extracts from an experimental rodent diabetic model. Additionally, novel human troponin-I phosphorylation sites were detected (Thr_119_, Thr_123_). Accelerated AGE modification of troponin-C was evident *in vitro* with hexose sugar exposure. This study provides the first demonstration of the occurrence of cardiac troponin complex AGE-modifications. These irreversible AGE modifications are situated in regions of the troponin complex known to be important in myofilament relaxation, and may be of particular pathological importance in the pro-glycation environment of diabetic cardiomyopathy.

## Introduction

The cardiac troponin complex facilitates cardiomyocyte contraction via dynamic interaction between the troponin subunits and the thin myofilaments, actin and tropomyosin. Troponins control the positioning of tropomyosin on the actin filament to regulate myosin-actin cross-bridge formation in response to Ca^2+^. The trimeric complex comprises troponin-C, which binds Ca^2+^ in low affinity binding sites during systolic Ca^2+^ influx; troponin-I, which inhibits the actin-myosin interaction and is tightly regulated by phosphorylation; and troponin-T, which anchors the troponin complex into the thin filament structure^1^. Post-translational modification of the troponin subunits is an important mechanism for mediating modulation of cardiomyocyte contractility in health and disease^2^, and novel reports of citrullination^3^, O-GlcNAcylation^4,5^, S-nitrosylation^6,7^, and phosphorylation^8–11^ sites on troponins have emerged in recent years. Post-translational modification of cardiac proteins for biomarker evaluation is also gaining interest^12–14^. To date, only dynamic post-translational modifications have been investigated, as a mode of reversible signaling regulation of the troponin complex. Susceptibility of troponins to irreversible post-translational modifications such as advanced glycation end-product (AGE) formation, has not been previously evaluated.

Troponin mutations are detected in many inherited cardiomyopathies^15^, providing evidence that small changes in the troponin protein sequence may have major implications for cardiac function. Both Ca^2+^ sensitizing and Ca^2+^ desensitizing troponin mutations have been identified, associated with either a hypertrophic/restrictive cardiomyopathy or a dilated cardiomyopathy phenotype respectively^15^. Similarly, modified troponin 3-dimensional structure due to the presence of glycated amino acid residues would be expected to contribute to alterations in Ca^2+^ sensitivity and could underlie acquired cardiopathologies such as heart failure and/or diabetic cardiomyopathy. Surprisingly, no studies to date have investigated this proposition - perhaps reflecting a presumption that the relatively short half-life of troponins (3–5 days)^16^ would preclude a functional impact of a reputedly slow ‘permanent’ glycation modification (conventionally considered to be of importance in the context of more stabilized macroprotein entities). Yet emerging evidence suggests that intracellular cardiac proteins with normally high turnover rates are indeed modified by glycation. Mass spectrometry studies have shown that sarcoplasmic reticulum Ca^2+^ ATPase and ryanodine receptor proteins are present in AGE modified states *in vivo* in diabetic rat hearts^17–19^, despite half-lives of 5–8 days^20^. The mechanism and time-course of intracellular AGE-modification is not well established, and presence of glycated proteins considered to be relatively high turnover may reflect AGE-induced delayed protein degradation and/or an environment of accelerated AGE formation.

AGEs are formed by non-enzymatic free sugar attachment to proteins and these adducts are converted through oxidation into fixed moieties which have potential to confer protein deformation and rigidity^21^. A physiological role for AGEs is unknown. Conflicting hypotheses have been posed that AGEs may act as either signals for protein degradation^22^ or inhibitors of protein degradation^23^. A growing body of evidence suggests that AGEs are primarily a form of environment-induced pathological protein damage with no known enzymatic processes for addition or removal. Although extracellular AGE-crosslinking of myocardial collagen is well-described^24^, few studies have investigated the role of AGE formation on intracellular proteins.

The rationale for investigation of troponin-AGE involvement in diabetic cardiomyopathy etiology is compelling. Not only do diabetic cardiomyocytes exhibit the pro-glycation environment of increased AGE precursors and oxidative stress, but increased exposure to fructose sugar, a potent glycation agent, may also play a role. An understanding of cardiomyocyte fructose actions is emerging^25,26^, and high myocardial fructose levels (up to 60-fold elevated) in diabetic rat hearts has been reported, likely driven by upregulation of the sorbitol pathway^27^. In type 2 diabetic rats, myocardial levels of Nε-carboxymethyllysine (CML), a common AGE modification, are correlated with impaired left ventricular relaxation^28^. In particular AGE-modification of troponin proteins has potential to impair the trimeric complex operation and contribute to cardiomyocyte dysfunction in pathological settings.

The goal of this study was to investigate human cardiac troponins (TnC, TnI and TnT), seeking evidence of endogenous AGE modifications using mass spectrometry screening techniques. As an outcome of AGE discovery our aim was to link the amino acid residue modification sites to functional domains in the protein sequence, and thus obtain biological insight relating to potential cardiac functional impact of these irreversible post-translational modification types. Our studies provide the first evidence that all three troponin subunits exhibit endogenous AGE-modification and demonstrate that human cardiac troponin-I is particularly susceptible to glycation events, occurring at amino acid sites of functional importance. Two novel phosphorylation sites (Thr_119_ and Thr_123_) on human cardiac troponin-I have been identified. Additionally, susceptibility of human cardiac troponin-C to accelerated AGE formation under *in vitro* conditions of high glucose and fructose exposure was established.

## Results

The investigative strategy firstly involved analyses of human-derived troponin subunits to identify occurrence of all glycated residues. This was followed by experimental confirmatory analyses of troponin glycation in cardiac tissue extract of control and diabetic rodents. Then an exploration of the positioning of glycation sites relative to phosphorylation sites detected was undertaken. Next, we evaluated the identified glycated residues in relation to known functional domains of each troponin. Finally, we performed experiments to assess residue susceptibility to glycation in a pro-glycation *in vitro* environment.

### AGE modification of human cardiac troponins

**Figure 1:**
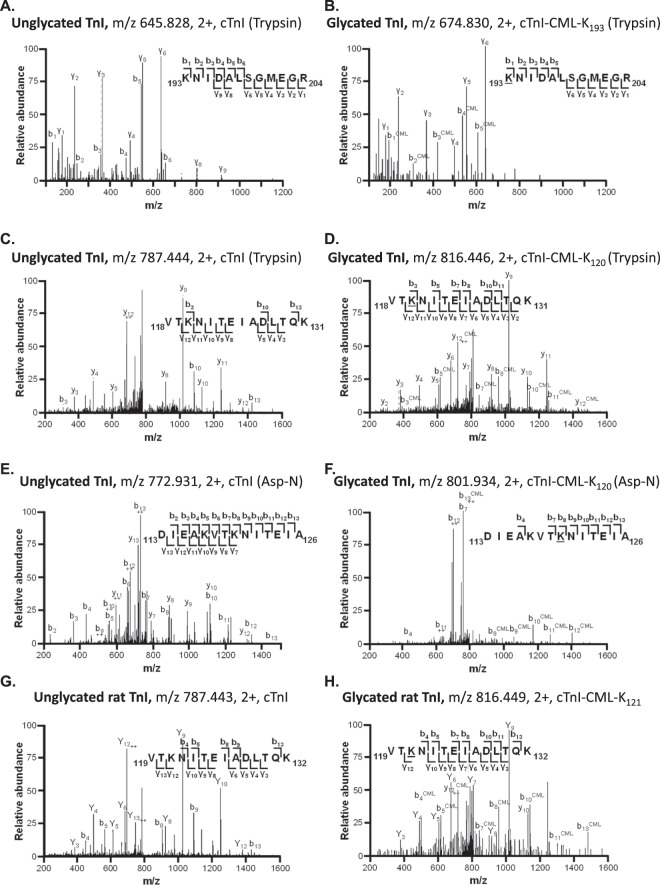
Novel AGE modification sites on human cardiac troponin-I (TnI). (**A**) MS/MS spectrum of trypsin-digested TnI unglycated peptide 193–204 (m/z 645.828). (**B**) MS/MS spectrum of trypsin-digested TnI peptide 193–204 with CML modification (+29 m/z = +58 Da) of Lys_193_ (m/z 674.830), confirmed by 4 peptides. (**C**) MS/MS spectrum of trypsin-digested TnI unglycated peptide 118–131 (m/z 787.444). (**D**) MS/MS spectrum of trypsin-digested TnI peptide 118–131 with CML modification (+29 m/z = +58 Da) of Lys_120_ (m/z 816.446), confirmed by 23 peptides. (**E**) MS/MS spectrum of AspN-digested TnI unglycated peptide 113–126 (m/z 772.931). (**F**) MS/MS spectrum of AspN-digested TnI peptide 113–126 with CML modification (+29 m/z = +58 Da) of Lys_120_ (m/z 801.934), confirmed by 7 peptides. (**G**) MS/MS spectrum of trypsin-digested rat TnI unglycated peptide 119–132 (m/z 787.443) corresponding to human TnI unglycated peptide 118–131. (**H**) MS/MS spectrum of trypsin-digested rat TnI peptide 119–132 with CML modification (+29 m/z = +58 Da) of Lys_121_ (m/z 816.449) corresponding to human TnI Lys_120_. The b-ions denote N-terminal ions and y-ions denote C-terminal ions. CML, N ε-carboxymethyl-lysine.

To determine AGE modification sites on human cardiac troponins, liquid-chromatography tandem mass spectrometric analysis (LC-MS/MS) of trypsin-digested purified human cardiac troponin-C, troponin-I and troponin-T was performed. All AGE modifications detected above identity threshold are detailed in Table 1. Troponin-C exhibited only 2 modified residues, CML modification of Lys_6_, and Lys_39_. Troponin-I exhibited numerous glycated residues: 13 amino acid residues modified by AGEs were identified. Lys_36_, Lys_50_, Lys_58_, Lys_117_, Lys_120_, Lys_131_, Lys_164_, Lys_183_ and Lys_193_ were modified by CML, Lys_120_ was also modified by Nε-carboxyethyllysine (CEL), and Arg_79_, Arg_148_, Arg_162_ and Arg_204_ were modified by methylglyoxal-derived hydroimidazolone (MG-H). Exemplar spectra for two of the troponin-I peptides are shown in Fig. 1 each in unglycated and glycated forms – Lys_193_ (Fig. 1A,B) exhibited low detection frequency and Lys_120_ (Fig. 1C,D) exhibited high detection frequency as is evident in Table 1. An annotated expansion of Fig. 1A,B is provided in the Supplementary Information (Fig. S1) to allow for closer comparison and inspection of unglycated versus glycated peptide spectra.

**Table 1:** Identification results of glycated human cardiac troponin proteins by LC-MS/MS analysis.

Protein	Peptide Sequence	Protease	Glycated Residue	AGE	# Peptide Observations	Calculated Mol. Mass	Observed m/z	Observed Mol. Mass	Error ppm	Identity Score	Expect	Charge
TnC	_1_MDDIYKAAVEQLTEEQKNEFK_21_	Trypsin	K_6_	CML	4	2586.2159	863.0802	2586.2187	1.07	59	1.5E-03	+3
							863.0800	2586.2180	0.82	56	3.0E-03	+3
							863.0797	2586.2173	0.54	43	6.4E-02	+3
							1301.1200	2600.2254	−2.35	62	5.7E-04	+2
	_22_AAFDIFVLGAEDGCISTKELGK_43_	Trypsin	K_39_	CML	1	2398.1726	1200.1000	2398.1854	5.35	45	3.5E-02	+2
TnI	_28_AYATEPHAKK_37_*	Trypsin	K_36_	CML	1	1172.5826	587.2987	1172.5829	0.26	53	2.0E-03	+2
	_46_KLQLKTLLLQIAK_58_	Trypsin	K_50_	CML	20	1567.0072	523.3400	1566.9982	−5.77	41	2.5E-03	+3
							784.5100	1567.0054	−1.13	40	2.8E-03	+2
							523.3432	1567.0078	0.36	59	3.7E-05	+3
							523.3427	1567.0061	−0.70	72	3.2E-07	+3
							523.3435	1567.0087	0.94	58	7.4E-06	+3
							523.3426	1567.0059	−0.81	21	4.0E-02	+3
							784.5104	1567.0063	−0.57	50	5.5E-05	+2
							523.3429	1567.0069	−0.23	35	1.6E-03	+3
							523.3430	1567.0070	−0.11	47	1.0E-04	+3
							784.5110	1567.0075	0.21	50	5.3E-05	+2
							523.3432	1567.0078	0.36	52	3.6E-05	+3
							784.5115	1567.0084	0.75	60	5.7E-06	+2
							784.5095	1567.0045	−1.74	70	6.2E-07	+2
							784.5104	1567.0063	−0.57	51	4.2E-05	+2
							523.3428	1567.0065	−0.46	33	2.9E-03	+3
							523.3431	1567.0076	0.24	33	2.8E-03	+3
							523.3426	1567.0059	−0.81	60	5.4E-06	+3
							784.5103	1567.0060	−0.80	45	1.6E-04	+2
							784.5104	1567.0063	−0.57	68	9.4E-07	+2
							523.3432	1567.0078	0.36	37	1.1E-03	+3
	_51_TLLLQIAKQELER_63_	Trypsin	K_58_	CML	3	1611.9195	806.9685	1611.9224	1.80	72	4.8E-05	+2
							806.9700	1611.9254	3.67	58	1.1E-03	+2
							806.9679	1611.9212	1.06	65	4.0E-05	+2
	_75_ALSTRCQPLELAGLGFAELQDLCR_98_*	Trypsin	R_79_	MG-H	1	2771.3734	693.8500	2771.3709	−0.92	58	8.9E-04	+4
	_113_DIEAKVTKNITEIA_126_	AspN	K_117_	CML	2	1601.8512	801.9330	1601.8515	0.22	48	9.9E-04	+2
							801.9338	1601.8531	1.21	34	2.0E-02	+2
	_113_DIEAKVTKNITEIA_126_	AspN	K_120_	CML	7	1601.8512	801.9337	1601.8528	0.98	48	8.6E-04	+2
							801.9339	1601.8532	1.29	31	4.2E-02	+2
							801.9301	1601.8457	−3.44	33	3.7E-02	+2
							801.9337	1601.8528	0.98	39	7.1E-03	+2
							801.9330	1601.8514	0.15	42	4.0E-03	+2
							801.9326	1601.8507	−0.31	37	1.2E-02	+2
							801.9349	1601.8553	2.59	34	2.3E-02	+2
	_118_VTKNITEIADLTQK_131_	Trypsin	K_120_	CML	23	1630.8777	816.4459	1630.8773	−0.28	70	7.5E-05	+2
							816.4462	1630.8779	0.10	73	3.4E-05	+2
							816.4464	1630.8781	0.25	68	9.9E-05	+2
							816.4463	1630.8780	0.17	68	1.0E-04	+2
							816.4464	1630.8782	0.29	64	5.4E-04	+2
							816.4500	1630.8854	4.72	69	1.3E-04	+2
							816.4416	1630.8686	−5.61	53	7.9E-03	+2
							816.4500	1630.8854	4.72	65	3.5E-04	+2
							816.4500	1630.8854	4.72	69	1.5E-04	+2
							816.4400	1630.8654	−7.54	57	3.3E-03	+2
							544.6342	1630.8808	1.86	46	3.8E-02	+3
							816.4457	1630.8769	−0.50	61	5.4E-04	+2
							816.4459	1630.8772	−0.35	57	1.4E-03	+2
							816.4467	1630.8788	0.62	95	2.3E-07	+2
							816.4456	1630.8766	−0.73	79	8.3E-06	+2
							816.4458	1630.8771	−0.43	75	2.3E-05	+2
							816.4501	1630.8857	4.89	46	2.0E-02	+2
							816.4450	1630.8755	−1.40	77	1.8E-05	+2
							816.4465	1630.8785	0.47	79	9.7E-06	+2
							816.4491	1630.8836	3.61	52	4.5E-03	+2
							816.4453	1630.876	−1.10	92	5.8E-07	+2
							544.6327	1630.8762	−0.92	44	2.4E-02	+3
							816.446	1630.8775	−0.13	65	2.2E-04	+2
			K_120_	CEL	1	1644.8934	549.3000	1644.8782	−9.26	50	1.3E-02	+3
TnI	_121_NITEIADLTQKIFDLR_136_	Trypsin	K_131_	CML	2	1947.0313	650.0189	1947.0350	1.92	77	2.4E-05	+3
							650.0189	1947.0348	1.82	53	6.0E-03	+3
	_147_VRISADAMMQALLGAR_162_*	Trypsin	R_148_	MG-H	1	1755.9124	586.3126	1755.9161	2.11	45	3.3E-02	+3
	_149_ISADAMMQALLGAR_162_*	Trypsin	R_162_	MG-H	1	1500.7428	751.3791	1500.7437	0.59	98	8.0E-08	+2
	_163_AKESLDLR_170_*	Trypsin	K_164_	CML	1	988.5189	495.2662	988.5179	−0.98	49	1.2E-02	+2
	_168_DLRAHLKQVKKEDTEKENREVG_189_*	AspN	K_183_	CML	1	2679.3940	670.8561	2679.3955	0.56	36	4.2E-02	+4
	_193_KNIDALSGMEGR_204_	Trypsin	K_193_	CML	4	1347.6452	674.8302	1347.6458	0.46	45	2.0E-02	+2
							674.8298	1347.6450	−0.18	61	7.6E-04	+2
							450.2224	1347.6455	0.20	56	2.3E-03	+3
							674.8287	1347.6428	−1.81	47	9.2E-03	+2
	_194_NIDALSGMEGR_204_	Trypsin	R_204_	MG-H	2	1215.5554	608.7853	1215.5560	0.56	57	1.1E-03	+2
							608.7849	1215.5553	−0.05	59	6.0E-04	+2
TnT	_105_MEKDLNELQALIEAHFENR_123_*	Trypsin	K_107_	CML	1	2357.1321	786.7200	2357.1382	2.58	45	2.7E-02	+3
	_124_KKEEEELVSLK_134_	Trypsin	K_125_	CML	3	1388.7398	695.3768	1388.7390	−0.60	43	4.0E-02	+2
							695.3778	1388.7411	0.89	45	1.6E-02	+2
							695.3771	1388.7396	−0.16	43	3.4E-02	+2
	_227_KVLAIDHLNEDQLR_240_	Trypsin	K_227_	CML	4	1720.9	861.4642	1720.9139	1.83	43	2.9E-02	+2
							861.4631	1720.9117	0.55	73	3.4E-05	+2
							861.4653	1720.9160	3.04	64	1.5E-04	+2
							861.4628	1720.9110	0.13	61	5.5E-04	+2

K, lysine; R, arginine; CML, carboxymethyllysine; CEL, carboxyethyllysine; MG-H, methylglyoxyl; ^*^Indicates single detection event data.

**Figure 2:**
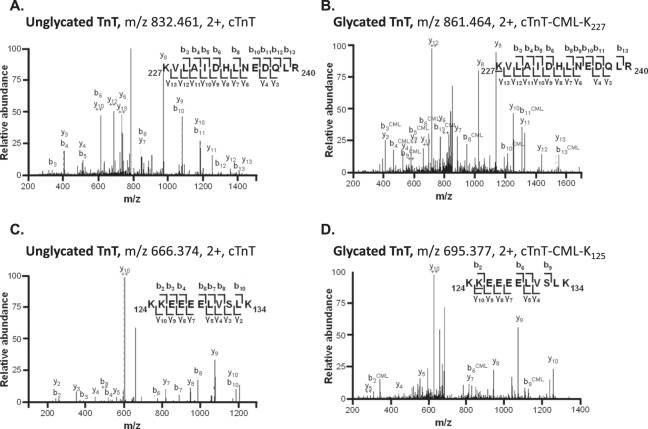
Novel AGE modification sites on human cardiac troponin-T (TnT). (**A**) MS/MS spectrum of trypsin-digested TnT unglycated peptide 227–240 (m/z 832.461). (**B**) MS/MS spectrum of trypsin-digested TnT peptide 227–240 with CML modification (+29 m/z = +58 Da) of Lys_227_ (m/z 861.464), confirmed by 4 peptides. (**C**) MS/MS spectrum of trypsin-digested TnT unglycated peptide 124–134 (m/z 666.374). (**D**) MS/MS spectrum of trypsin-digested TnT peptide 124–134 with CML modification (+29 m/z = +58 Da) of Lys_125_ (m/z 695.377), confirmed by 3 peptides. The b-ions denote N-terminal ions and y-ions denote C-terminal ions. CML, N ε-carboxymethyl-lysine.

Given the abundance of glycation sites on troponin-I, the endoproteinase AspN was used to digest troponin-I at aspartate and cysteic acid residues to confirm that AGE detection was not dependent on trypsin cleavage at Lys and Arg residues. As for trypsin digestion, AspN-digested troponin-I exhibited CML modification of Lys_120_ in both unglycated and glycated form (Fig. 1E,F). AspN digestion also yielded evidence of 2 additional CML modification sites, Lys_117_ and Lys_183_, which were not detected with trypsin-digestion (Table 1). Exploratory investigations were undertaken to establish evidence of AGE occurrence in an *in vivo* experimental setting. Crude tissue extract was prepared from myocardium of control and type1 diabetic rodents (8 weeks duration, streptozotocin treated). AGEs were detected on Lys_59_, Lys_121_, Lys_194_ of troponin-I derived peptides from diabetic but not control samples (Table S1). These sites represent the rodent equivalent of corresponding AGE modification sites identified on human troponin-I (Lys_58_, Lys_120_, Lys_193_). For these troponin-I AGE modifications, which could only be detected in the diabetic myocardium, selected exemplar spectra of unglycated and glycated troponin-I (Lys_121_) are provided in Fig. 1G,H. Human troponin-T exhibited 3 AGE modification sites with CML modification detected at Lys_107_, Lys_125_ and Lys_227_. Figure 2 shows spectra for those residues where multiple glycation detection events were observed. Thus, Lys_227_ and Lys_125_ in both unglycated and glycated form are shown in Fig. 2A–D. Three AGE modification sites were detected on troponin-T isolated from control and diabetic rat homogenate (Lys_109_, Lys_202_, Lys_228_) confirming two of the corresponding AGE-modification sites identified on human troponin-T (Lys_107_, Lys_227_). In addition, one new AGE-modification site was detected at Lys_202_ (Table S1).

### Identification of novel phosphorylation sites on human cardiac troponin-I using LC-MS/MS

**Figure 3:**
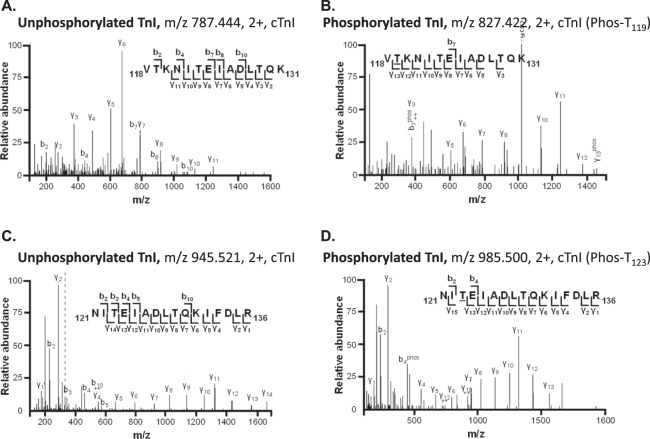
Novel phosphorylation sites on human cardiac TnI. (**A**) MS/MS spectrum of trypsin-digested TnI unphosphorylated peptide 118–131 (m/z 787.444). (**B**) MS/MS spectrum of trypsin-digested TnI peptide 118–131 with phosphorylation (+40 m/z = +80 Da) of Thr_119_ (m/z 827.422), confirmed by 3 peptides. (**C**) MS/MS spectrum of trypsin-digested TnI unphosphorylated peptide 121–136 (m/z 945.521). (**D**) MS/MS spectrum of trypsin-digested TnI peptide 121–136 with phosphorylation (+40 m/z = +80 Da) of Thr_123_ (m/z 985.500). The b-ions denote N-terminal ions and y-ions denote C-terminal ions. Phos, phosphorylation.

LC-MS/MS analysis of trypsin-digested human cardiac troponin-I peptides identified 7 phosphorylation sites (Table 2 and Supplement Information). Analogous unmodified peptides were detected for all reported phosphorylation sites. Phosphorylation sites previously reported in the literature (Ser_23/24_, Thr_51_, Ser_150_, Ser_166_, Ser_199_) were confirmed in the purified human cardiac troponin-I samples in this study. The previously-reported dual Ser_42/44_ troponin-I phosphorylation events were not detected in these samples, likely due to the small size of the tryptic peptide containing these residues. Novel unreported troponin-I phosphorylation sites (Thr_119_ and Thr_123_) were also identified. Spectra of these troponin-I peptides in the presence and absence of phosphorylation at Thr_119_ and Thr_123_ are shown in Fig. 3A–D. Interestingly, these novel phosphorylation sites are in close proximity to the CML- and CEL-modification site Lys_120_ shown in Table 1 and Fig. 1D,F, suggesting that AGE modification at Lys_120_ has the potential to interfere with regulatory phosphorylation sites. All spectra where novel phosphorylation occurrence was detected on troponin-I (Thr_119_ and Thr_123_) are provided in the Supplementary Information.

**Table 2:** Identification of phosphorylation sites on human cardiac troponin-I using LC-MS/MS analysis.

Peptide Sequence	Phosphorylated Residue	# Phosphorylated Peptides	Calculated Mol. Mass	Observed m/z	Observed Mol. Mass	Error ppm	Identity Score	Expect	Charge
_21_RRSSNYR_27_	S_23/24_	2	1097.4168	549.7153	1097.4160	−0.76	44	3.5E-03	+2
				549.7153	1097.4160	−0.71	41	6.9E-03	+2
_46_KLQLKTLLLQIAK_58_	T_51_	3	1588.9681	795.4868	1588.9591	−5.65	54	7.7E-05	+2
				530.6605	1588.9596	−5.32	46	4.2E-04	+3
				795.4870	1588.9595	−5.37	32	1.2E-02	+2
_51_TLLLQIAKQELER_63*_		1	1633.8804	817.9432	1633.8719	−5.17	50	2.3E-03	+2
_118_VTKNITEIADLTQK_131_	T_119_	3	1652.8386	827.4221	1652.8296	−5.41	79	7.6E-06	+2
				827.4241	1652.8336	−3.03	78	1.1E-05	+2
				827.4225	1652.8305	−4.87	86	6.7E-07	+2
_121_NITEIADLTQKIFDLR_136*_	T_123_	1	1968.9921	985.5000	1968.9854	−3.39	74	2.2E-05	+2
_146_RVRISADAMMQALLGAR_162_	S_150_	2	1937.9692	647.0000	1937.9782	4.62	54	3.6E-03	+3
				646.9972	1937.9697	0.24	59	8.3E-04	+3
_147_VRISADAMMQALLGAR_162_		6	1797.8630	891.9428	1781.8711	−0.17	76	2.4E-05	+2
				594.9632	1781.8678	1.65	48	9.6E-02	+3
				594.9639	1781.8698	1.22	79	1.3E-05	+3
				891.9424	1781.8703	0.95	44	3.9E-02	+2
				891.9436	1781.8727	2.54	65	3.2E-04	+3
				594.9645	1781.8715	1.92	53	5.3E-03	+2
_149_ISADAMMQALLGAR_162_		3	1526.6986	764.3565	1526.6985	−0.08	95	1.3E-07	+2
				764.3567	1526.6988	0.15	92	1.2E-07	+2
				764.3565	1526.6985	−0.09	62	1.3E-04	+2
_163_AKESLDLR_170_	S_166_	2	1010.4797	506.2469	1010.4792	−0.48	54	2.8E-03	+2
				506.2469	1010.4793	−0.45	41	5.0E-02	+2
_194_NIDALSGMEGR_204*_	S_199_	1	1257.5060	629.7601	1257.5056	−0.31	33	6.4E-02	+2

S, serine; T, threonine; ^*^Indicates single detection event data.

### Location of AGE- and phosphorylation-modification sites on the human cardiac troponin complex

**Figure 4:**
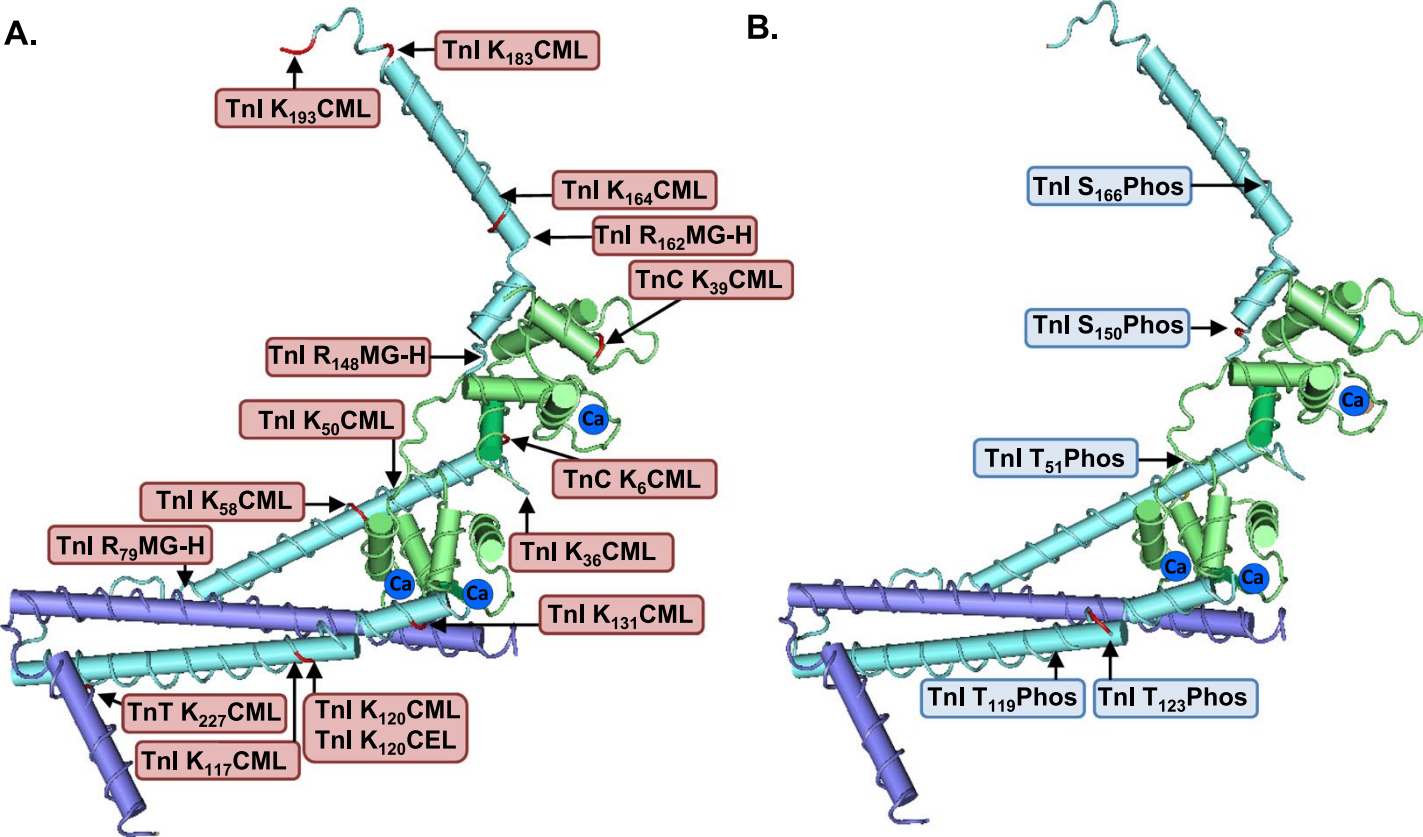
Amino acid residue location of identified AGE and phosphorylation modification sites on human cardiac troponin complex proteins. (**A**,**B**) 3-dimensional structure of cardiac troponin-C (TnC, green), troponin-I (TnI, aqua) and troponin-T (TnT, mauve). Modification sites are shown in red. Location of Ca^2+^ binding pockets indicated by dark blue circles. TnI R_204_MG-H, TnI S_23/24_Phos, TnI S_199_Phos, TnT K_107_CML and TnT K_125_CML are not shown as the 3-dimensional structure is not resolved for the C-terminal domain of TnI and N-terminal of TnT. CML, N ε-carboxymethyl-lysine; CEL, N ε-carboxyethyl-lysine; MG-H, methylglyoxal-derived hydroimidazolone; Phos, Phosphorylation.

**Figure 5:**
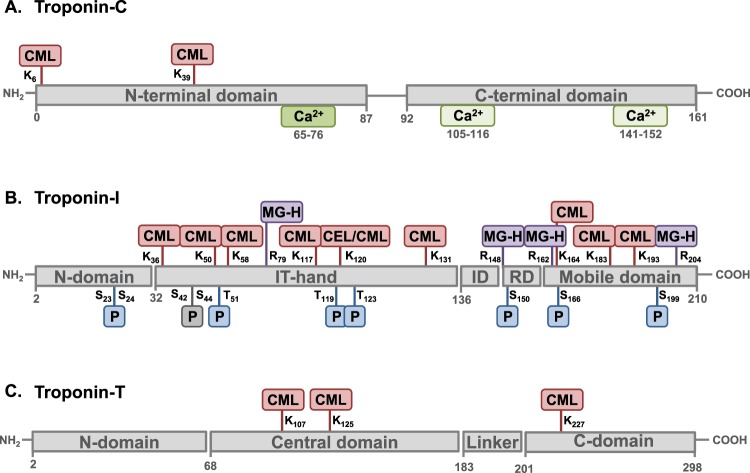
Domain structure of human troponin complex proteins indicating identified AGE and phosphorylation modification sites detected by LC-MS/MS. (**A**) Domain structure of human cardiac troponin-C. Ca^2+^ binding sites are depicted in green: dark green, high-affinity Ca^2+^ binding site; light green, low affinity Ca^2+^ binding sites. (**B**) Domain structure of human cardiac troponin-I. ID, inhibitory domain; RD, regulatory domain. Detected phosphorylation sites in blue, known but not detected site in grey. (**C**) Domain structure of human cardiac troponin-T. CML, N ε-carboxymethyl-lysine; CEL, N ε-carboxyethyl-lysine; MG-H, methylglyoxal-derived hydroimidazolone; P, phosphorylation.

As depicted in Fig. 4A, several AGE-modification sites identified by LC-MS/MS on the troponin subunits are located within protein-protein interaction regions of the 3-dimensional structure of the troponin complex. Figure 4B presents the identified phosphorylation sites on the 3-dimensional structure of the troponin complex. Figure 5A depicts the domain structure of the troponin subunits showing that CML-modified troponin-C amino acids, Lys_6_ and Lys_39_, are located within the N-terminal domain. Figure 5B illustrates that several glycated residues are situated adjacent to phosphorylated residues in the troponin-I sequence. Specifically, CML-modified Lys_50_ and Lys_58_ are in close proximity to the phosphorylation site Thr_51_. CEL/CML-modified Lys_120_ is located adjacent to the novel phosphorylation site Thr_119_ and near to Thr_123_. Glycation sites within the mobile domain (MG-H-Arg_162_, CML-Lys_164_, CML-Lys_183_, CML-Lys_193_, and MG-H-Arg_204_) may have important implications for movement of this domain in response to Ca^2+^ and for phosphorylation of Ser_166_ and Ser_199_. Figure 5C illustrates that the CML-modified Lys_107_, Lys_125_ and Lys_227_ are located within the central- and C-domain of troponin-T. Thus, glycated troponin residues may modify interaction of the troponin subunits and influence phosphorylation-mediated signaling regulation of the troponin complex.

### *In vitro* high glucose or fructose incubation of human cardiac troponin-C accelerates AGE-formation

**Figure 6:**
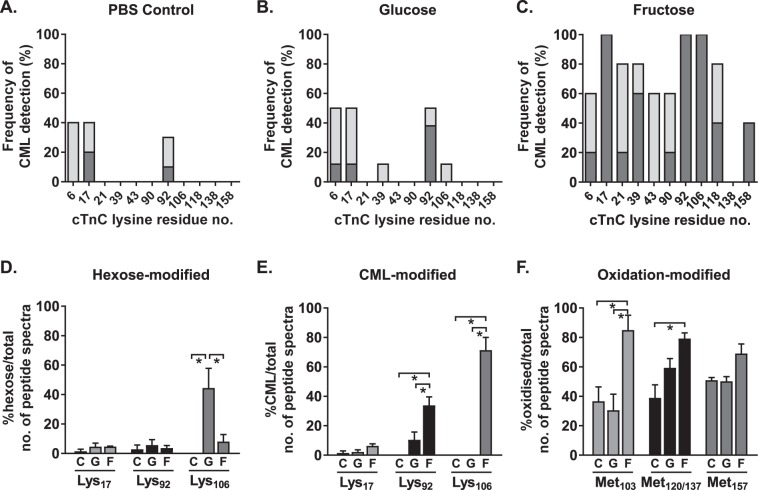
*In vitro* exposure to glucose and fructose increases AGE-modification of human cardiac troponin-C (TnC). (**A**–**C**) Percentage of technical replicates with detection of a CML modification at lysine residues of purified human cardiac TnC using LC-MS/MS (total 5–7 technical replicates per condition). Dark grey: modified, detection above identity threshold; light grey: modified, detection below identity threshold. CML, carboxymethyllysine. (**D**–**F**) Number of TnC MS/MS peptide spectra with hexose, CML, or oxidation modifications as a percentage of the total number of TnC MS/MS spectra detected for a given peptide (n = 5 replicates/group). Criteria for identifying a modification: above homology threshold and expect value < 0.5. Data presented as mean ± SEM. One-way ANOVA, Bonferroni post-hoc tests, *p < 0.05.

To experimentally examine whether glycation of human cardiac troponin could be promoted by hexose incubation *in vitro*, and to evaluate site-specific ‘glycation-vulnerability’, human purified cardiac troponin-C was incubated with 2 M glucose or 2 M fructose for 7 days at 37 C. Of the three purified troponin preparations, only troponin-C is soluble in the absence of urea. Thus troponin-C is suitable for *in vitro* investigations as urea-induced carbamylation of potential AGE sites (Lys and Arg) is avoided. Control (phosphate-buffered saline, PBS), glucose- and fructose-incubated troponin-C samples were trypsin-digested and processed by LC-MS/MS. Given that the conventional view is that the formation of AGEs can take weeks to months, we hypothesized that hexose attachments (AGE initiation) or Amadori products (AGE-precursors) might be evident. We anticipated that it would be unlikely that AGE formation (e.g. CML) would be observed within this time period. Surprisingly, CML-modification of troponin-C was observed after only 7-days of incubation with glucose or fructose (Fig. 6A–C). To identify the human cardiac troponin-C amino acid residues with the highest susceptibility to AGE formation, the LC-MS/MS fragmented peptide spectra were analyzed for 5–7 replicates per group (PBS control, 2 M glucose or 2 M fructose) and the percentage of replicates with AGE-modified amino acid residues are presented in Fig. 6A–C. The frequency of CML detection on amino acid residues of troponin-C was slightly higher in glucose-incubated samples, relative to PBS control (for residues Lys_6_, Lys_17_, Lys_39_, Lys_92_ and Lys_106_), although not all CML-modified residues reached the identity threshold of detection (Fig. 6A,B). Fructose-incubated troponin-C had the highest frequency of replicates with CML-modified amino acid residues, and detection of CML-modified Lys_17_, Lys_92_ and Lys_106_ was observed in 100% of replicates (Fig. 6C).

Spectral counting was performed to semi-quantify the hexose-, CML- and oxidation-modifications observed on troponin-C in response to glucose and fructose exposure. Troponin-C peptides containing the most frequently detected modified amino acid residues (Lys_17_, Lys_92_ and Lys_106_) were counted and presented as the number of modified peptides as a percentage of the total number of peptides (modified and unmodified). Comparisons were made between incubation groups for any single modification site. No differences in hexose modification were observed between PBS control, glucose or fructose incubated troponin-C for Lys_17_ and Lys_92_ sites (Fig. 6D). In contrast, hexose-modified Lys_106_ was significantly higher in the glucose-incubated troponin-C samples relative to fructose (44.4 ± 13.4% vs 8.0 ± 4.9%, p < 0.05, Fig. 6D). Considering all glycation sites, less than 2% of troponin-C peptides were modified by CML in the PBS control group. Glucose incubation increased CML modification to 10.5 ± 5.2% at the Lys_92_ site (p < 0.05, Fig. 6E). Fructose-incubated troponin-C exhibited the greatest extent of CML modification with 33.9 ± 5.8% of Lys_92_ and 71.3 ± 8.7% of Lys_106_ containing peptides detected with a CML-related mass shift (p < 0.05, Fig. 6E). Oxidation of methionine residues in troponin-C peptides was prominent in all groups. Relative to PBS incubated troponin-C, oxidation significantly increased in fructose incubated troponin-C derived Met_103_, and Met_120/137_ containing peptides (p < 0.05, Fig. 6F).

Collectively these findings suggest that glucose exposure may promote the initiation of AGE modification of troponin-C evidenced by increased hexose attachments, while fructose may accelerate AGE formation beyond the hexose initiation step evidenced by higher CML modification coincident with higher oxidation of methionine residues (and lower pre-AGE formation hexose attachments). These findings also confirm that AGE formation on troponin-C can occur within 7 days, a finding consistent with the observation that intracellular proteins of relatively short half-life exhibit AGE modification *in vivo*.

## Discussion

This study provides the first evidence that all subunits of the human cardiac troponin complex are modified by endogenous AGE formation. Our data demonstrate that human cardiac troponin-I is particularly susceptible to AGE modification, with 13 glycation sites detected, many of which are located adjacent to phosphorylation sites or within protein-protein interaction regions. The findings show that some, but not all, troponin lysine and arginine residues exhibit AGE modification indicating that particular residues are more vulnerable to glycation than others. Analysis of troponins isolated from rodent hearts revealed troponin-I derived from diabetic but not control rat myocardium exhibited AGE modification. These studies have also revealed preliminary evidence for two novel phosphorylation sites on human cardiac troponin-I (Thr_119_ and Thr_123_). *In vitro* experiments with human cardiac troponin-C have demonstrated that AGE formation is promoted in a hexose-enhanced incubation setting, and that fructose in particular is an AGE accelerant. Collectively these findings provide new evidence that the human cardiac troponin complex is a potential target for glycation events at numerous sites, including locations of key importance for cardiomyocyte mechano-operation. Such molecular events may be of particular relevance in pathological settings, including diabetic cardiomyopathy, where hexose intracellular milieu is disturbed and reactive oxygen species levels are elevated.

During each cardiomyocyte activation cycle a complex sequence of molecular conformational changes involving the trimeric troponins underpins the conversion of an electrically-driven Ca^2+^ signal to a contractile outcome for the cardiac pump. Ca^2+^ binds to troponin-C and troponin-I changes conformation to reveal the myosin binding site on actin, thus allowing cross-bridge formation to occur. Dissociation of Ca^2+^ from troponin-C initiates contractile cycle relaxation by allowing troponin-I to reposition and re-instate the suppression of myosin-actin interaction. AGE-modification of troponins has considerable capacity to disturb the rapid cyclic dynamic movements of the troponin subunits, altering Ca^2+^-troponin-C association/dissociation kinetics, and disrupting protein-protein interactions. As apparent in Figs 4 and 5, the potential conformational impact of an AGE modified residue on troponin complex subunit positioning, and the spatial relationships between AGE- and phosphorylation responsive residues, provides for substantial pathophysiologic substrate.

All AGE modifications detected on troponin-I are located within protein binding sites, thus it is likely that binding of troponin-I to other troponins in the complex, and/or to myofilaments, would be affected. In the present study, multiple CML and MG-H sites were detected on troponin-I in the C-terminal tropomyosin positioning element. Mutations in this region elicit myofilament Ca^2+^ sensitizing effects and predispose to hypertrophic and/or restrictive cardiomyopathy phenotypes, where systolic function is maintained but diastolic function declines^29–32^. AGE modification within these regions may predispose for similar effects, consistent with increased Ca^2+^ sensitivity and diastolic dysfunction in diabetic cardiomyopathy. Based on the extensive literature relating to troponin-I mutation effects, the case for future studies designed to assess the potential modulation of contractile performance by troponin-I glycation is compelling.

CML is detected in the troponin-I binding site for the C-lobe of troponin-C, the secondary actin/tropomyosin binding site and the mobile domain of troponin-I. The positive charge of Lys and Arg residues is neutralized by AGE modification, potentially reducing hydrophilicity of the troponin-I domains^33,34^. Previous studies have identified charged amino acid residues as crucial elements of the troponin subunits for ensuring effective binding between all three subunits and for interaction with actin/tropomyosin^35–37^. AGE-induced neutralization of Lys and Arg in binding sites between the three subunits may decrease the structural stability of the complex and lead to uncoupling of Ca^2+^ binding and actomyosin cross-bridge formation. AGE-modification of the secondary actin/tropomyosin binding domain on troponin-I may also interfere with its role in Ca^2+^ dissociation and actin inhibition^38^. Neutralization of critical charged Lys and Arg residues by AGEs located in the mobile domain of troponin-I may be associated with slowed and/or incomplete myofilament relaxation and increased diastolic cardiomyocyte stiffness.

Several troponin-I domains are intrinsically disordered^34,39,40^, thus their conformation is highly responsive to the structural influence of post-translational modifications^41,42^. Key signaling kinases, protein kinase C, protein kinase A, protein kinase G, and p21 activated kinase 3, phosphorylate troponin-I resulting in modulation of myofilament Ca^2+^ sensitivity and contractility in response to various stimuli^10,43^. In the present study, five AGE-modified troponin-I residues are identified within two amino acids of a phosphorylation site, thus it could be expected that AGE-modification may interfere with signaling regulation of the troponin complex. In addition to confirmation of well-known and recently identified phosphorylation sites on troponin-I (Ser_23/24_, Thr_51_, Ser_150_, Ser_166_, Ser_199_), this study provides preliminary evidence for two novel phosphorylation sites (Thr_119_, Thr_123_). Interestingly, these sites span the identified robust CML and CEL modification of Lys_120_. Given that these residues are located within the troponin-I/troponin-T α-helical coiled coil^1^, phosphorylation and AGE-modification at these sites may be important for troponin complex structural backbone stability and tropomyosin anchoring. Further analysis of the Thr_119_ and Thr_123_ phosphorylation sites is now warranted, including identification of the relevant kinase(s) and characterization of functional implications.

Troponin-C and troponin-T exhibited fewer endogenous AGE-modification sites than troponin-I. CML-modification was detected on 2 troponin-C amino acid residues (Lys_6_ and Lys_39_) and 3 troponin-T amino acid residues (Lys_107_, Lys_125_ and Lys_227_). Spectral counting methods revealed that the overall percentage of troponin-C peptides with CML modification was very low (<2%) but increased after 1 week of *in vitro* hexose exposure (~70% for Lys_106_ exposed to fructose). Glucose incubation appeared to promote the initiation of AGE modification of troponin-C evidenced by increased hexose attachments on Lys_106_, while fructose accelerated AGE formation beyond the hexose initiation step, evidenced by lower hexose attachments but higher CML modification coincident with higher oxidation of methionine residues. Small changes in AGE modification of cardiac troponins would be expected to influence protein structure and function - a recent study demonstrated that a 6% change in Ser_199_ pseudo-phosphorylation status of troponin-I is sufficient to alter myofilament Ca^2+^ sensitivity^8^. These findings now prompt further investigation. As noted above, due to differing ionization efficiencies between peptides comparison could not be made between AGE modification sites. Development of site-specific quantitation methodology is required for further comparative analysis of *in vitro* and *in vivo* AGE accumulation. Further exploration of troponin-C AGE vulnerability is required, including more extensive proteolytic studies involving AspN. Additionally, systematically assessing the role of ambient hexose and oxidative stress conditions on AGE formation will yield important pathophysiologic insight. The *in vitro* hexose conditions utilized in this study are clearly artificial, contrived to achieve strongest evidence of AGE-site susceptibility. Similarly, while the atmospheric incubation environment utilized provides a starting point, a range of conditions should be investigated and the influence of subcellular compartmentalization, concentrating hexose and reactive oxygen species considered.

The 7 day *in vitro* incubation findings support the contention that AGE formation is more rapid than generally understood (previously estimated as weeks to months)^44^, further corroborated by the extensive observation of AGE modifications of troponin proteins considered to have short life times. AGE formation may delay protein turnover - in diabetic states where pronounced and sustained intracellular metabolic stress is evident, proteins and any accompanying AGE precursors (hexose, methylglyoxal, glyoxal, α- dicarbonyls) may persist longer in cytosolic locations due to dysfunction of intracellular degradative processes such as the glyoxalase^45^ and ubiquitin-proteasome system^46^ or autophagy pathways^47^. It could be expected that AGE modification of intracellular cardiac proteins may be promoted in the pro-glycation environment of altered hexose flux and oxidative stress in diabetic cardiomyocytes. Based on the ‘proof of concept’ data generated in this investigation, detailed evaluation of site-specific AGE modification accumulation and associated cardiomyocyte functional deficits in human and rodent diabetic heart tissues is warranted.

## Limitations

While robust, the findings reported in this investigation involve aspects of inherent bias – both sampling and technical. As a ‘proof of concept’ study, the evaluation of purified proteins involved samples derived from a limited number of human subjects. More extensive studies are required to evaluate a range of tissue samples obtained from diabetic and non-diabetic patients. To maximize identification of AGE modification sites present in the samples evaluated in this study, technical replicates were utilized, and this may confer false positive risk. Conversely, a relatively conservative algorithm was employed to establish detection events of AGE-modified proteins, with a high threshold ‘identity’ requirement. This approach may produce an AGE ‘under-detection’ bias, the extent of which can only be determined by further investigation. These considerations highlight the imperative for further exploration of the role of troponin AGE-modification in myocardial pathophysiology.

## Conclusions

This is the first study to demonstrate that all three human cardiac troponin subunits can exhibit AGE modification. Human cardiac troponin-I appears most susceptible to glycation, with thirteen modified amino acid residues detected. Given the importance of troponin-I in mediating the Ca^2+^-sensitive troponin complex conformational change with each cardiac cycle, it is likely that obstructive irreversible glycation modifications would elicit effects on cardiomyocyte function. Troponin-I AGE modifications were detected in regions of protein-protein interaction, adjacent to phosphorylation sites and in key regions of conformational change. The findings presented in this study generate new biological insight relating to potential cardiac functional impact of irreversible post-translational modification of cardiac troponins and provide the platform for new *in vivo* investigations to advance understanding of the functional outcomes of cardiac troponin AGE modification. In particular, investigations into the relative AGE abundance on cardiac troponins in a diabetic setting, where hexose flux disturbance and oxidative stress coincide to generate a pro-glycation environment, will be an important priority. Glycation of cardiac troponins may represent a novel mechanism underlying cardiomyocyte relaxation defects in diabetic cardiomyopathy, and offer an important new target for therapeutic intervention. Further studies of AGE occurrence in cardiac disease states are now warranted.

## Methods

### Animal model

Sprague Dawley rats purchased from the Animal Resource Centre (WA, Australia) were housed in polypropylene cages in a temperature-controlled room at a 12:12 hour light and dark cycle at the Biomedical Sciences Animal Facility at the University of Melbourne and provided access to water and standard chow ad libitum for the duration of the experiment. Type 1 diabetes was induced in male rats at 8 weeks of age via a single 55 mg/kg tail vein injection of streptozotocin (STZ; Sigma) with weight and blood glucose monitored for 8 weeks. Hyperglycemia was present in STZ-treated animals from 2 days post injection and at 8 weeks post-injection, all STZ rats exhibited blood glucose >30 mmol/L vs. control animals 6.45 ± 0.16 mmol/L. At 8 weeks after diabetes induction, rats were euthanized by cervical dislocation (isoflurane anaesthesia), hearts were excised, and left ventricles were dissected and frozen at 80 °C. Frozen tissue was homogenized in 100 mM Tris·HCl, 5 mM EGTA, and 5 mM EDTA (Sigma-Aldrich) buffer containing protease and phosphatase inhibitors (Roche, Switzerland). STZ and Control hearts (N = 4 animals/group, n = 2 technical replicates/ heart) were used to generate *in vivo* rat TnI glycation data. All animals were cared for in accordance with the Australian Code of practice for the Care and Use of Animals for Scientific Purposes (NHMRC, 2013). All procedures conducted as part of this study were approved by the Animal Ethics Committee of The University of Melbourne (Approval Numbers:, 1613861).

### Preparation of cardiac troponins for LC-MS/MS acquisition

Troponin-C, troponin-I and troponin-T were purified from human cardiac tissue harvested from non-myocardial infarction human donors by Life Diagnostics (PA, USA). Human troponin-I, C and T samples were each typically purified from a single donor heart. Each aliquot acquired from a troponin I (n = 10), C (n = 5) or T (n = 5) sample was considered a technical replicate for these studies. Troponin-I and C were purified by urea extraction of cardiac muscle, ion-exchange chromatography followed by hydrophobic affinity chromatography (TnC) or troponin-C affinity chromatography (TnI). Troponin-T was purified by salt extraction and fractionation of cardiac muscle followed by ion-exchange chromatography. The purity was confirmed by the manufacturer to be >98% for troponin-I and troponin-T, and >95% for troponin-C. For analysis of endogenous AGE- and phosphorylation-modifications, coomassie-stained troponin bands were excised from SDS-PAGE gels, diced into 1 mm^3^ cubes, destained for 2 hours with 50% acetonitrile in 50 mM triethylammonium bicarbonate buffer (TEAB), dehydrated with 100% acetonitrile for 30 minutes, reduced with 10 mM tris(2-carboxyethyl)phosphine (TCEP, Life Technologies, VIC, Australia) for 45 minutes at 55 °C and alkylated with 50 mM iodoacetamide (proteomics grade Life Technologies, Vic, Australia) for 30 minutes, shielded from light. After alkylation, gel pieces were washed 3 times in 50 mM TEAB for 10 minutes, dehydrated with 100% acetonitrile for 30 minutes and digested overnight in sequencing-grade trypsin (25 µg/mL, Sigma-Aldrich, MO USA) or sequencing-grade AspN (40 μg/mL; Promega, WI USA) in 25 mM TEAB at 37 °C with shaking. Samples were then acidified with 1% formic acid and centrifuged at 16,000 g for 10 minutes. The supernatant (20 μL) containing digested peptides was then transferred to Exigen vials, and stored at 4 ^o^C pending LC-MS/MS acquisition^48,49^.

To determine whether particular lysine or arginine residues are more ‘glycation-vulnerable’ purified human cardiac troponin-C (0.137 µg/µL, Life Diagnostics, PA, USA) was incubated in PBS alone or containing 2 M glucose or 2 M fructose at 37 ^o^C sealed under ambient atmospheric conditions for 7 days. Short incubation pilot experiments showed that hexose attachments formed within 2 days in 2 M glucose or 2 M fructose conditions so a longer 7 day incubation was selected to assess the formation of AGE-modifications. Following the incubation period, troponin-C (4 μg) was diluted with 50 mM TEAB, and reduced with 5 mM TCEP (Life Technologies, Vic, Australia) at 60 ^o^C for 10 minutes. Samples were digested in sequencing-grade trypsin (5ug/ml; 1:50 protease: protein ratio; Sigma-Aldrich, MO, USA) at 37 ^o^C overnight in TEAB with shaking followed by acidification in 1% v/v formic acid. Following centrifugation (16,000 g, 10 minutes), the supernatant (20 µL) containing digested peptides was transferred to Exigen vials, and stored at 4 ^o^C pending LC-MS/MS acquisition^48^.

### LC-MS/MS acquisition and analysis

Digested peptides were separated by liquid chromatography using a nanoflow-reversed-phase-HPLC (Ultimate 3000 RSLC; Dionex, CA, USA). The digested peptides were loaded onto an Acclaim Pepmap nano-trap column (C18, 100 Å, 75 μm × 2 cm; Dionex, CA, USA) with 3% acetonitrile/0.1% formic acid (5 μL/min, 6 min) and switched in-line to an Acclaim Pepmap RSLC analytical column (C18, 100 Å, 75 μm × 50 cm; Dionex, CA, USA) with solvent A, 0.1% formic acid and solvent B, 100% acetylnitrile/0.1% formic acid (gradient: 3–20% B for 95 min, 20-40% B for 10 min, 40–80% B for 5 min, and 80% B for 5 min). All spectra were acquired in positive mode with full scan MS with spectra range m/z 375–1400 at 70,000 resolution, automatic gain control target of 3e6 ions, maximum accumulation time of 50 ms and a lock mass of 445.120024 m/z. The 15 most intense peptide ions with charge states ≥2–5 were isolated (1.2 m/z window) and fragmented with normalized collision energy of 30 and spectra acquired in positive mode MS with 17,500 resolution, automatic gain control target of 1e5 ions, and maximum accumulation time of 100 ms. Mass spectra of peptide ion fragments were acquired using a Thermo Scientific OrbiTRAP Elite MS/MS, or a Thermo Scientific Q-Exactive plus mass spectrometer.

Mass spectra were collected and compiled using the MSILE2 platform (Bio21, University of Melbourne) and bioinformatics analysis performed using the MASCOT Pipeline (Matrix Science, London, UK)^50^. Peptide spectra were compared against theoretical *in silico* peptide signatures in the Mass Spectrometry protein sequence Database (MSDB; 53,659,159 sequences) to identify sequence coverage of troponin-C, troponin-I and troponin-T. MSDB database trawling was restricted to i) Homo sapien (purified human troponin-C, troponin-I and troponin-T samples), and ii) peptides were produced via trypsin proteolysis (C-term lysine and arginine cleavage only) or AspN proteolysis (N-terminal aspartic acid and cysteine cleavage only) with up to 2 missed cleavages per peptide permitted. Peptide scores were assigned based on the number of ions detected and matched with theoretical ions, and signal intensity relative to background noise. Peak match tolerance was 0.2 Da. An identity threshold score was assigned to represent a peptide score with 95% confidence. Criteria used to determine a protein match included: combined score >1000, 5 or more unique peptides detected, peptide scores greater than identity threshold scores and false discovery rate <5%. Peptide identity scores were determined using a ‘probability based scoring’ algorithm to evaluate the observed match between the experimental data and the database sequence^50^. The corresponding identity threshold calculation was based on the number of peptides that fell within the precursor mass tolerance window and the significance threshold that was selected (0.05). Probing for post-translational modification sites were limited to hexose, CML, CEL, MG-H, and phosphorylation (MASCOT bioinformatic searching platform). Methionine oxidation was selected as a variable modification. Carbamidomethyl cysteine was selected as a fixed modification as it is a result of iodoacetamide incubation during sample preparation (capping of cysteine residues to inhibit re-formation of disulphide bonds following TCEP reduction). Modifications were confirmed by manually matching theoretical ion masses to spectra with known mass shifts relating to hexose +162 Da, CML + 58 Da, CEL + 72 Da, and MG-H + 54 Da. Phosphorylation sites were identified as mass shifts of +80 Da, and/or by neutral loss scanning of phosphoric acid (−98 Da). Doubly charged peptides will present with half the mass shift e.g.(+29 for CML) and triply charged peptide ions will present a third of the expected mass shift e.g. (+19.3 for CML). Quantification of hexose, AGE- and oxidation-modification of troponin-C was performed by counting modified peptide spectra normalized to total (modified and unmodified) peptide counts (above homology threshold) for a particular peptide of interest. Amino acid residues were numbered as presented in the Uniprot database (UniProtKB - P63316 (TNNC1_HUMAN), P19429 (TNNI3_HUMAN), P45379 (TNNT2_HUMAN)) where full sequence data may be obtained.

### Statistical analyses

Data are presented as mean ± sem. Statistical analyses were performed using GraphPad Prism V7.0 (GraphPad, CA, USA). Data were analyzed by one-way ANOVA with Bonferroni post-hoc tests where appropriate. A p-value of < 0.05 was considered statistically significant.

## Electronic supplementary material

Supplementary information

## Data Availability

Full spectral dataset will be made available online (Scientific Data https://www.nature.com/sdata/).
