# Supplementary material for: Cardiac troponins may be irreversibly modified by glycation: novel potential mechanisms of cardiac performance modulation
Source: Sci Rep. 2018 Oct 31;8:16084. doi: 10.1038/s41598-018-33886-x (PMC6208411; doi:10.1038/s41598-018-33886-x)
Supplement: Supplementary file 1 — Supplementary information [file 41598_2018_33886_MOESM1_ESM.pdf]

Supplementary Information

Cardiac troponins may be irreversibly modified by glycation: novel potential mechanisms of cardiac performance modulation

Johannes V Janssens, Brendan Ma, Margaret A Brimble,
Jennifer E Van Eyk, Lea MD Delbridge[#], Kimberley M Mellor[#]

Figure S1. Expanded comparison of unglycated and glycated peptide spectra

Upper panel. Expanded MS/MS spectrum of trypsin-digested TnI unglycated peptide 193-204 (m/z 645.828). **Lower panel.** Expanded MS/MS spectrum of trypsin-digested TnI peptide 193-204 with CML modification (+29m/z = +58Da) of Lys_{193} (m/z 674.830). The panels have been positioned to allow comparison of unglycated to glycated fragment ion peak distribution. The blue circles in the lower panel indicate fragment ion peaks that have been shifted by a CML adduct. In particular b-ions 1-5 are shifted to the right in the lower panel in comparison with the upper panel. The b-ions denote N-terminal ions and y-ions denote C-terminal ions. CML, N ε-carboxymethyl-lysine.

Table S1. Identification results of glycated rat cardiac troponin proteins by LC-MS/MS analysis.

List of all detected troponin-I or T derived peptides exhibiting glycation. TnI-Lys_{59}, Lys_{121}, Lys_{194} in rat is corresponding to TnI-Lys_{58}, Lys_{120}, Lys_{194} in human respectively. TnT-Lys_{109}, Lys_{202}, Lys_{228} in rat is corresponding to TnT-Lys_{107}, Lys_{200}, Lys_{227} in human respectively.

Figure S1. Expanded comparison of unglycated and glycated peptide spectra

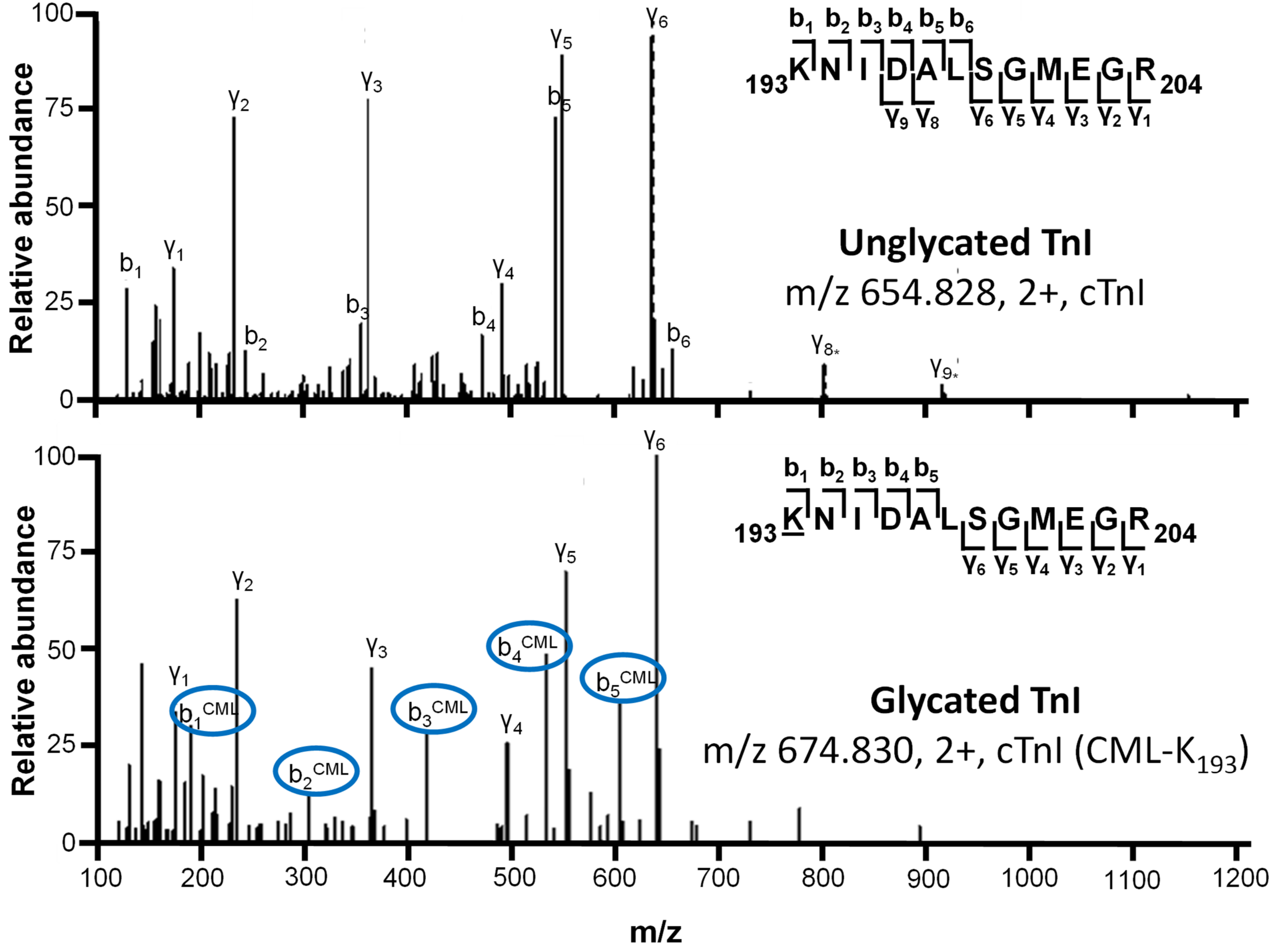

Table S1. Identification results of glycated rat cardiac troponin proteins by LC-MS/MS analysis.

Protein	Peptide Sequence	Protease	Glycated Residue	AGE	# Peptide Observations	Calculated Mol. Mass	Observed m/z	Observed Mol. Mass	Error ppm	Identity Score	Expect	Charge	Animal model
TnI	$_{52}\text{TLMLQIA}\underline{\text{K}}\text{QEMEREAEER}_{69}$	Trypsin	K_{59}	CML	1	2262.0983	755.0470	2262.1192	9.21	53	9.0E-04	3+	STZ
	$_{119}\text{VT}\underline{\text{K}}\text{NITEIADLTQK}_{132}$	Trypsin	K_{121}	CML	1	1630.8777	816.4492	1630.8838	3.69	40	7.3E-03	2+	STZ
	$_{194}\underline{\text{K}}\text{NIDALSGMEGR}_{205}$	Trypsin	K_{194}	CML	1	1347.6452	674.8310	1347.6474	1.63	47	1.6E-03	2+	STZ
	$_{194}\underline{\text{K}}\text{NIDALSGM}_{(\text{Ox})}\text{EGR}_{205}$	Trypsin	K_{194}	CML	1	1363.6402	682.8284	1363.6423	1.58	36	1.7E-02	2+	STZ
TnT	$_{106}\text{RME}\underline{\text{K}}\text{DLNELQTLIEAHFENR}_{125}$	Trypsin	K_{109}	CML	2	2543.2438	636.8244	2543.2685	9.72	38	3.0E-02	4+	CON
							636.8227	2543.2617	7.05	40	1.9E-02	4+	CON
	$_{107}\text{ME}\underline{\text{K}}\text{DLNELQTLIEAHFENR}_{125}$	Trypsin	K_{109}	CML	6	2387.1427	597.7981	2357.1633	8.64	58	3.1E-04	4+	CON
							796.7272	2387.1598	7.16	75	5.9E-06	3+	STZ
							597.7980	2387.1629	8.47	56	4.2E-04	4+	STZ
							796.7281	2387.1427	8.30	89	2.3E-07	3+	CON
							796.7291	2387.1655	9.55	67	4.0E-05	3+	STZ
							796.7285	2387.1637	8.80	69	2.1E-05	3+	CON
	$_{189}\text{ALSNMMHFGGYIQ}\underline{\text{K}}\text{AQTER}_{207}$	Trypsin	K_{202}	CML	1	2239.0514	747.3641	2239.0705	8.53	41	1.5E-02	3+	CON
	$_{228}\underline{\text{K}}\text{VLAIDHLNEDQLR}_{241}$	Trypsin	K_{228}	CML	1	1720.9108	861.4706	1720.9266	9.23	52	27.4E-04	2+	CON

K, lysine; R, arginine; CML, carboxymethyllysine; Ox, oxidation; STZ, Streptozotocin induced diabetic rat; CON, Control (Sprague Dawley rat)
